# Link Prediction of Green Patent Cooperation Network Based on Multidimensional Features

**DOI:** 10.3390/e28020155

**Published:** 2026-01-30

**Authors:** Mingxuan Yang, Xuedong Gao, Yun Ye, Junran Liu

**Affiliations:** School of Economics and Management, University of Science and Technology Beijing, Beijing 100083, China; d202310506@xs.ustb.edu.cn (M.Y.); m202210985@xs.ustb.edu.cn (Y.Y.); u202341159@xs.ustb.edu.cn (J.L.)

**Keywords:** patent cooperation network, link prediction, multidimensional features, green patent

## Abstract

The regional green patent cooperation network describes the structural characteristics of regional collaborative innovation, and the link prediction of the network can anticipate the overall evolution trend, as well as help organizations identify potential partners for technology collaboration. This paper proposes a link prediction model based on multidimensional features, which integrates prediction indicators of node features, path features, and content features. In the model, the entropy weight method is employed to integrate various node similarity indicators, the heterogeneous influence of intermediate links and nodes is incorporated to fully emphasize the issue of heterogeneous paths, and the content similarity feature indicator based on patent text topic analysis integrates multiple distance similarity metrics. To improve prediction accuracy, the Grey Wolf Optimizer (GWO) method is adopted to determine the optimal weights for the three-dimensional indicators. The comparative experimental results show that the multidimensional prediction model can improve prediction accuracy significantly. Finally, the proposed prediction model is applied to forecast the green patent cooperation network in the Beijing-Tianjin-Hebei region of China, and the prediction results are discussed based on the distribution of agent types and regional distribution.

## 1. Introduction

The patent cooperation network depicts the cooperative relationships formed by various patent applicants through joint patent filings. It constitutes a complex network encompassing inter-enterprise collaboration, collaboration between enterprises and universities, as well as collaboration between enterprises and research institutions. As an important manifestation of collaborative innovation among innovation agents, studies on the patent cooperation network can reveal the characteristics and pathways of collaborative innovation [1,2]. In recent years, to promote the innovation-driven effect of developed regions on their surrounding areas, the Chinese government has formulated strategies for regional collaborative innovation development. Through measures such as resource allocation, industrial restructuring, and tax incentives, the government aims to boost innovation cooperation among sub-regions within a major regional cluster. Typical examples of such collaborative development regions include the Beijing-Tianjin-Hebei region, the Yangtze River Delta region, and the Pearl River Delta region. Against this policy backdrop, numerous scholars have conducted research on intra-regional patent cooperation networks to evaluate the effectiveness and dynamics of regional collaborative innovation. Meanwhile, in the process of regional collaborative innovation, green technology innovation has attracted growing attention due to its significance for regional ecology, environmental protection, and sustainable development. Therefore, it is of great practical significance to study regional green patent cooperation networks, analyze their structural characteristics, and reveal their evolutionary trends through link prediction. On the one hand, predicting the future development trends of green technology innovation cooperation within a region enables the early identification of network structural deficiencies, the evaluation of the driving effect of collaborative innovation, and the optimization of policies for green technology collaborative innovation. On the other hand, link prediction can assist patent applicants in identifying potential partners and facilitating the realization of collaborative innovation.

Although extensive research on link prediction has been conducted based on knowledge cooperation networks, few studies have specifically focused on link prediction for regional green patent cooperation networks. Against this backdrop, this paper aims to construct a regional green patent cooperation network and perform link prediction to uncover its evolutionary trends. At the methodological level, the primary challenge lies in selecting appropriate prediction indicators and constructing an effective prediction model to enhance prediction accuracy. Existing link prediction studies have employed a wide array of indicators, such as those based on node similarity, path similarity, and content similarity, with each category encompassing multiple specific indicators. Scholars typically combine one or more types of indicators to improve prediction accuracy [3,4,5]. Building on existing research, this paper proposes a novel hybrid model based on multidimensional features. Compared with previous related studies, the proposed model exhibits the following distinctive features: (1) For indicators within the same category, a multi-indicator coupling method based on the entropy weight method is introduced. (2) The heterogeneity of intermediate links and intermediate nodes in the network is simultaneously incorporated into path features. (3) Content similarity, node similarity, and path feature indicators are all integrated into the prediction model, and a method for calculating the optimal weights of these three indicator categories is proposed.

The remainder of this paper is organized as follows. Section 2 reviews the related research. Section 3 elaborates on the construction method of the prediction model. Section 4 presents prediction experiments conducted on a green patent dataset of the Beijing-Tianjin-Hebei region and provides a comparative analysis of multiple prediction models. Section 5 performs a predictive network analysis of link prediction results for the Beijing-Tianjin-Hebei green patent cooperation network and offers policy recommendations for the development of green technology collaborative innovation in the region.

## 2. Related Work

From the perspective of research objects, the regional green patent cooperation network falls within the category of patent cooperation networks. From the perspective of research methods, link prediction is categorized under the theoretical study of complex networks. Therefore, the review of related work will be organized around two aspects: patent cooperation networks and link prediction. Specifically, studies on patent cooperation networks cover three sub-themes: patent cooperation networks, green patent cooperation networks, and regional green patent cooperation networks. Studies on link prediction include three major types of prediction indicators: node-based, path-based, and semantic-based, as well as the relevant applications of combinations of these indicators in the prediction of knowledge cooperation networks.

(1) Research on patent cooperation networks

Utilizing patent applicant data to construct patent cooperation networks has long been a hot topic in the field of collaborative innovation. Tsay et al. [1] constructed a global patent cooperation network in the field of artificial intelligence and analyzed the evolution characteristics of this network. Chen et al. [2] collected patent cooperation application data of different types of organizations in China from 2007 to 2015 and constructed an inter-organizational patent cooperation network. Liu [6] studied the relevant patent network of Tsinghua University in China and analyzed the important role of universities in collaborative innovation. Liu et al. [7], based on the patent collaborative application data of the National Intellectual Property Administration, applied complex network theory and social network analysis methods to study the collaborative network of China’s wind energy industry. Mei et al. [8] constructed a collaborative innovation network based on collaborative patent and paper data from the Beijing-Tianjin-Hebei region from 2010 to 2016 to explore the characteristics and efficiency of innovation cooperation among cities in this region.

Although patent data have been widely used in the study of the characteristics of collaborative innovation networks, there are few studies on green patent data and green innovation. Liu et al. [9] utilized Chinese patent data and the patent classification numbers provided by the IPC Green List to construct a green patent cooperation network from 2007 to 2017. They analyzed the characteristics of this network from multiple perspectives, including time, region, and spillover effect. Zhou et al. [10] collected green patent data of the Yangtze River Delta urban agglomeration from 2000 to 2016 and constructed a patent cooperation network, analyzing the evolution model of green collaborative innovation in the Yangtze River Delta region. Fan et al. [11] analyzed the spatial correlation network of China’s green innovation using the gravity model and social network analysis model, and the results showed that there was a spatial effect in the green innovation correlation network of China. It was found that regions with strong green technological innovation capabilities did not have a significant impact on the green innovation of other provinces, while regions with medium innovation strength might play a crucial role in the green innovation linkage network. Wang et al. [12] investigated the spatial correlation network structure of green innovation efficiency in the Yangtze River Delta region, revealing that the region’s green innovation efficiency in the Yangtze River Delta region was extremely unbalanced and the spatial network correlation density was low. Bai et al. [13] proposed a framework for determining the impact of multiple relationship networks on green innovation. They employed text mining to construct multiple relationship networks, identified green patents from massive patent data through content analysis, and then utilized fuzzy set Qualitative Comparative Analysis (fsQCA) to examine the equivalent impact paths of green innovation. In the above studies on the structure of green patent cooperation networks, although relatively rich results have been achieved, they primarily focus on analyzing network characteristics, influencing factors, and evolution patterns.

(2) Research on link prediction

Link prediction in social networks refers to identifying missing connections within the network based on existing relationships or predicting whether unconnected nodes will establish connections in the future [3]. For enterprises engaging in innovation cooperation, selecting the appropriate R&D partners is crucial in determining whether collaborative innovation can succeed and enhancing organizational performance [14]. Therefore, identifying possible innovation partners through link prediction is also a hot topic in recent research on cooperative networks. The most commonly used indicators for innovation cooperation network link prediction can currently be divided into three categories: node-based, path-based, and semantic-based [15,16].

The prediction algorithm based on node features is based on the common neighbors of nodes and uses the structural similarity matrix of network nodes to represent the similarity between nodes, such as Common Neighbor Indicator (CN), Salton Indicator, Jaccard Indicator, Hub Promoted Index (HPI), LHN-I Indicator, Adam-Adar Indicator (AA), and Resource Allocation Indicator (RA) [15,17,18,19]. These algorithms primarily rely on local information and are logically straightforward to implement, offering high computational efficiency. Chen et al. [2] introduced eight commonly used node similarity indicators to predict cooperative relationships based on the patent application data in 2016 and found that the CN index was particularly suitable for organizations’ decision-making on choosing unfamiliar partners in patent cooperation. Zhang et al. [20] compared 10 common indicators based on node features and found that the AA index had the best prediction accuracy in the patent cooperation application network of the Guangdong-Hong Kong-Macao Greater Bay Area. Shi et al. [21] used the entropy weight method and integrated the four most accurate prediction indicators based on node features to develop a new indicator, which proved more accurate in predicting cooperation between scientific and technological entities in the Beijing-Tianjin-Hebei region.

The prediction methods based on path features utilize network paths for prediction, such as Katz index [22], LP index [23,24], and LHN-II [17]. Among these methods, the Katz index makes predictions based on the global path information between two nodes, but it has a high time complexity in calculation [22]. Zhou et al. [23,24] proposed the Local Path (LP) index, which only incorporates paths of length 2 and 3 into the prediction, balancing accuracy and time complexity, but it does not consider the issue of path heterogeneity. In recent years, prediction algorithms based on path heterogeneity features have continued to develop. Zhu et al. [25] fully utilized the degree information of intermediate nodes in similarity calculation and proposed the Significant Path (SP) index. Empirical experiments on twelve different real-world networks show that this index outperforms mainstream link prediction baseline methods. Zhu et al. [26] believe that the connectivity of intermediate nodes also affects path heterogeneity, and based on this, they proposed the semi-local index NSI (Neighbor Set Information). Some studies on innovation cooperation have also integrated path indicators with other indicators, Liu and Sun [27], who fused the path-based feature indicators with the similarity of research interests, and Wang et al. [28], who fused the CN, RA, Jaccard, AA based on node features with the Katz index based on path features to predict potential patent partners.

With the rapid development of innovation networks, some researchers have found that traditional prediction algorithms based on node and path features are no longer sufficient to meet the prediction requirements of cooperative networks, and have therefore turned to applying text semantic features for the prediction of cooperative networks. For example, Chuan et al. [16] proposed a link prediction algorithm based on Latent Dirichlet Allocation (LDA) topic modeling from the perspective of content similarity, utilizing cosine similarity to calculate the similarity between topics and replacing the original prediction algorithm that relies on network structure features. Jeon et al. [29] identified potential partners with the required technology by mining the relationships between words in patent claims. Wang et al. [30] proposed a new algorithm that identifies R&D partners based on the similarity of subject–action–object semantics. Kang et al. [31] further proposed a method for selecting sustainable industry–university–research cooperation partners based on an LDA topic model.

In research on link prediction in cooperative networks, there is a trend of integrating indicators based on node, path, and semantic features. The integration methods consider both the semantic features of the cooperative content and the structural features of the cooperative network. For example, Park et al. [32] explored potential R&D partners through literature coupling and patent semantic analysis. Ding et al. [33] developed a method for mining potential partnerships based on the similarity of authors’ paper contents and the structure of the cooperative network.

(3) Review of related work

Given the dual importance of regional collaborative innovation and green technology innovation, it is highly necessary to conduct link prediction for regional green patent cooperation networks. However, a review of existing studies indicates that research on regional green patent cooperation networks has mainly focused on network characteristic analysis, spatial effects, and innovation pathways, whereas link prediction research on such networks remains underexplored. This research gap has motivated the present study. In addition, the current research on prediction indicators has covered a wide range of perspectives, yet there remain notable limitations in the application of these indicators to Patent Network Prediction (PNP), as shown in Table 1. First, integrating the network’s topological structure with semantic content similarity has emerged as a new trend in link prediction, but this approach has not yet been applied to the link prediction of regional green patent cooperation networks. Second, heterogeneous paths have not been fully considered, and few studies have correlated the specific topological structure of patent cooperation networks with the issue of path heterogeneity. Third, with regard to the combined prediction using multiple indicators, a unified indicator coupling method and an optimal weight calculation method are required. Therefore, how to integrate multidimensional prediction indicators into the prediction model and improve the accuracy of link prediction constitutes the primary problem to be addressed in conducting link prediction for regional green patent cooperation networks.

## 3. Research Method

**Figure 1:**
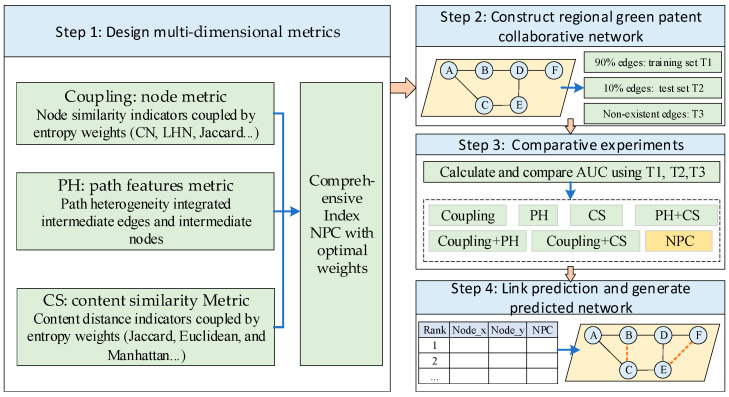
Method framework for this study.

To achieve link prediction for regional green patent cooperation networks, a comprehensive model based on multidimensional indicators is proposed. This model integrates predictive indicators from three dimensions: node features, path features, and content features. In terms of node features, the entropy weight method is employed to couple traditional node similarity indicators, enhancing the universality of the indicators. Regarding path features, the heterogeneous influences of intermediate links and intermediate nodes are integrated to fully emphasize the issue of heterogeneous paths. For content features, the LDA model is used to construct topic set vectors for nodes, and multiple similarity distances are integrated to calculate content similarity (CS). The methodological framework of the model construction is illustrated in Figure 1. In the framework, the network is composed by nodes and edges, and the letters in the node circle means the number of the patent applicants.

### 3.1. Predictive Indicator Based on Node Similarity Metric (Coupling)

The network constructed based on patent cooperation data can be represented as G = (N, E, W), where N represents the set of nodes in the network, E represents the set of edges in the network, W represents the edge weight, which is the number of collaborations of the entities, and |N| represents the total number of network nodes.

Link prediction considering node feature similarity holds that the higher the similarity of nodes, the greater the possibility of cooperation [15]. For example, the CN index refers to the number of common neighbors the two nodes share; the more likely the two nodes are to cooperate. Node similarity indicators have evolved over the years, and there are currently 10 commonly used indicators. The formulas are shown in Table 2. In the formula, $k_{x}$ represents the degree of node *x*, $\Gamma_{x}$ and represents the neighbor nodes of node *x*.

The prediction accuracy of each of the above indicators varies in different networks. Therefore, in this paper, the 10 node similarity indicators are coupled using the entropy weight method, and the constructed coupled indicator is denoted as Coupling, as shown in Formula (1):

(1) $$S_{xy}^{Coupling}=a_{1}S_{xy}^{CN}+a_{2}S_{xy}^{LHN}+a_{3}S_{xy}^{Jaccard}+a_{4}S_{xy}^{PA}+\ldots$$

Among them, $a_{i}$ represents the weight value based on the entropy weight method, $a_{i}\in \left[0,\;1\right]$. $S_{xy}^{Coupling}$ represents the similarity score of nodes *x* and *y* under the coupling index, $S_{xy}^{CN}$ represents the similarity score of nodes x and y under the common neighbor (CN) structure, and $S_{xy}^{LHN}$ is similar.

The entropy weight method draws on the concept of entropy in thermodynamics and uses information entropy to describe the amount of information of an event. In this paper, it represents the amount of information that each indicator accounts for in node features: the smaller the entropy value, the greater the dispersion degree of the indicator, and thus the greater its contribution to indicator ranking. For weight calculation using the entropy weight method, the first step is to perform sum standardization on each indicator, as shown in Formula (2).

(2) $$p_{ij}=\frac{R_{ij}}{\sum_{j=1}^{10}R_{ij}}\;$$

where *j* denotes the indicator number, which ranges from 1 to 10 in this paper; *i* denotes the node pair number. Secondly, calculate the information entropy value of each indicator for all node pairs, as shown in Formula (3).

(3) $$E_{j}=-\frac{1}{lnm}\sum_{i=1}^{m}p_{ij}{lnp}_{ij}\;$$

where *m* represents the total number of node pairs to be predicted. Since the entropy value is inversely proportional to the contribution, a positive relationship transformation is performed on the entropy value, as shown in Formula (4).

(4) $$a_{j}=\frac{1-E_{j}}{\sum_{j=1}^{n}\left(1-E_{j}\right)}\;$$

### 3.2. Predictive Indicator Based on Heterogeneous Path Metric (HP)

The path characteristics in the cooperation network can also accurately predict the cooperative relationship. However, when constructing the path characteristics, it is necessary to distinguish the influence of different paths on the prediction results, so as to further improve the prediction accuracy of the path characteristics. In this study, in order to distinguish the heterogeneity of the paths between two entities, the heterogeneity of the intermediate edges and the heterogeneity of the intermediate nodes are simultaneously introduced into the path characteristics.

Intermediate edge heterogeneity: For a link with multiple intermediate nodes, the larger the degrees of the two end nodes, the more dispersed their cooperation intentions. Thus, the less likely these two nodes will cooperate [26]. Here, the product of the degrees of the two end nodes is used to represent the link weight, that is, the heterogeneity of the intermediate edge. The link weight is negatively correlated with the cooperation probability. Therefore, for two nodes *x* and *y* in the network with a link between them, the influence of the link weight can be expressed as ${\left(k_{x}\;k_{y}\right)}^{-1}$. For the intermediate nodes between nodes *x* and *y*, all the intermediate nodes are represented by a set, denoted as $Z=\left(Z_{1},Z_{2},\ldots ,Z_{M}\right)$, and the total heterogeneity of the intermediate edge of this intermediate node set is denoted as $S_{xy}^{L}$, which is expressed by Formula (5):

(5) $$S_{xy}^{L}={\left(k_{x}\cdot k_{Z_{1}}\right)}^{-1}+\sum_{m=1}^{M-1}{\left(k_{Z_{m}}\cdot k_{Z_{m+1}}\right)}^{-1}+{\left(k_{Z_{M}}\cdot k_{y}\right)}^{-1}$$

Intermediate node heterogeneity: Even with the heterogeneity of edge connections, when the product of the degrees of the nodes in the intermediate links is equal, it still cannot effectively differentiate the impact of paths on cooperation. Therefore, further consider the heterogeneity of intermediate nodes as a weighted weight to enhance the discrimination of path heterogeneity. Intermediate node heterogeneity refers to the higher the connectivity between intermediate nodes, the more stable the connection between end nodes, and the higher the possibility of cooperation [25].

For node pairs *x* and *y* in the network that have at least one intermediate node, all the intermediate nodes are recorded as a whole Z, and the number of intermediate nodes is represented by |Z| = M. When M = 1, it is a second-order path, and its connectivity is represented as whether the intermediate node Z and the two neighboring nodes *x* and *y* can form a triangular ring. |$Z_{△}|$ represents the actual number of triangular rings formed by all the surrounding neighbors of Z, and |$Z_{\Lambda }|$ represents the number of all possible triangular rings that the surrounding neighbors of Z can form. Then, the second-order path heterogeneity score of the intermediate nodes Z of nodes *x* and *y* is recorded as Formula (6):

(6) $$S_{xy}^{N}=\frac{\left|Z_{△}\right|}{\left|Z_{\Lambda }\right|}$$

When M = 2, it becomes a third-order path, and its connectivity lies in whether the intermediate node set $Z=\left(Z_{1},Z_{2}\right)$ and the two neighboring nodes *x* and *y* can form a quadrilateral loop. At this time, the influence of the intermediate node set on similarity is similar to that when M = 1, that is, the actual number of quadrilateral loops formed is divided by the possible number of quadrilateral loops.

Considering the characteristic that the prediction accuracy decreases rapidly with the increase of path length, as well as the time complexity issue of the calculation process, this paper sets the number of intermediate nodes to M = 2. The heterogeneity path (HP) index $S_{xy}^{HP}$ based on the intermediate edges and intermediate nodes is constructed, and is expressed by Formula (7):

(7) $$S_{xy}^{HP}={\left(S_{xy}^{L}+S_{xy}^{N}\right)}_{M=1}+\alpha \cdot {\left(S_{xy}^{L}+S_{xy}^{N}\right)}_{M=2}$$

Among them, $S_{xy}^{HP}$ represents the heterogeneity score of nodes x and y based on the heterogeneity of intermediate edges and intermediate nodes, $S_{xy}^{L}$ represents the heterogeneity score of nodes *x* and *y* based solely on the heterogeneity of intermediate edges, $S_{xy}^{N}$ represents the heterogeneity score of nodes *x* and *y* based solely on the heterogeneity of intermediate nodes, and α is the weight value of the third-order path heterogeneity index, with $\alpha \in \left[0,\;1\right]$.

### 3.3. Predictive Indicator Based on Content Similarity Metric (CS)

In addition to the network structure information, the technical field of the patent applicant also has a significant impact on the cooperative relationship. Therefore, the content similarity between nodes needs to be introduced into the prediction model. The specific steps are as follows: The patent abstract is taken as the main content for text analysis. The LDA model is used to divide the patent abstract into themes. Then, vector similarity indicators such as the Jaccard coefficient, Euclidean distance, and Manhattan distance are utilized to calculate the content similarity of the patent, representing the degree of association of the applicant’s technical field.

Before applying the LDA model for topic classification, the optimal number of topics needs to be determined first. In this paper, the CV consistency score method is used to calculate the optimal number of topics K. Based on this, the LDA model is applied to calculate the topic distribution probability of each patent, and the topic with the highest probability value is selected as the topic of that patent. The calculation of probability is as shown in Formula (8).

(8) $$P\left(w|d\right)=P\left(w|t\right)\cdot P\left(t|d\right)$$

Among them, *P*(*w*|*d*) represents the probability of word *w* in document *d*, *P*(*w*|*t*) represents the probability of word *w* in a specific topic *t*, and *P*(*t*|*d*)represents the probability of document *d* in a specific topic *t*.

After obtaining the topic of each patent, the topic is assigned to each applicant entity of the patent, indicating that the research direction of this applicant includes this topic. After assigning the topics one by one, a topic set vector can be formed for each applicant, representing the complete set of research directions of this applicant. Finally, the patent content similarity between applicants is calculated based on the topic set vectors. To improve the prediction effect, six common vector similarity indicators were selected, as shown in Table 3. Based on the experimental results, the three indicators with the best prediction accuracy were selected, and then the entropy weight method was used for coupling.

The formula of similarity index for the coupled content is shown as follows:

(9) $$S_{xy}^{CS}={b_{1}S}_{xy}^{1}+b_{2}S_{xy}^{2}+{b_{3}S}_{xy}^{3}$$

Among them, $b_{1},b_{2},b_{3}$ represent the weight values based on the entropy weight method, and $b_{1},b_{2},b_{3}\in \left[0,\;1\right]$. $S_{xy}^{CS}$ represents the content similarity score of nodes *x* and *y* under the coupling index, while $S_{xy}^{1}\;$, $S_{xy}^{2}$, $S_{xy}^{3}$ are the top three vector similarity indicators.

### 3.4. Comprehensive Index NPC Based on Network Topology Structure and Content Similarity

By integrating the coupling node similarity index Coupling, the path heterogeneity index HP, and the content similarity index CS, a new link prediction index is constructed and denoted as NPC (Node & Path & Content index). Its calculation is as shown in Formula (10):

(10) $$S^{NPC}={x_{1}S}^{Coupling}+x_{2}S^{HP}+{x_{3}S}^{CS}$$

Among them, $x_{1},x_{2},x_{3}$ are the optimal weight values of each indicator obtained through mathematical programming. The mathematical model is as Formula (11):

(11) $$min:f=-AUC\left(S^{NPC}\right)=-AUC\left(x_{1}S^{Coupling}+x_{2}S^{HP}+x_{3}S^{CS}\right)\;S_{.}T_{.}\left\{\begin{array}{c} x_{1}+x_{2}+x_{3}=1\\ 0\leq x_{1},x_{2},x_{3}\leq 1 \end{array}\right.$$

Among them, ${AUC(S}^{NPC})$ represents the prediction accuracy of the NPC indicator, while $S^{NPC}$, $S^{Coupling}$, $S^{HP}$ and $S^{CS}$ are the similarity score matrices generated for the corresponding indicators.

## 4. Empirical Research

### 4.1. Data Collection and Organization

This paper selected the cooperative data of green invention patents in the Beijing-Tianjin-Hebei region from 2020 to 2023. The selected case is located in the Beijing-Tianjin-Hebei (BTH) region of China. Since 2014, this region has been designated as a national priority under the Beijing-Tianjin-Hebei Coordinated Development Strategy, whose connotation encompasses both collaborative innovation and green sustainable development within the region. Geographically, the region comprises Beijing, China’s capital city, and Tianjin, a directly administered municipality, both of which are surrounded by Hebei Province, a less economically developed area. The motivation behind the BTH Coordinated Development Strategy is to drive the integrated development of Tianjin and Hebei through the radiating effect of Beijing, covering such dimensions as talent sharing, industrial restructuring, resource transfer, and green development. Therefore, the selected case serves as a typical representative of regional green technology collaboration under China’s coordinated development policies, and thus holds notable representativeness and significance for research.

The data were sourced from the IncoPat global patent database. During the data search, the green patent classification number, applicant address, application time, number of applicants, applicant type, and business registration address were limited in the initial search formula. Among them, the green patent classification number was a patent classification number compiled based on the Chinese “International Patent Classification Table” and the “International Patent Classification (IPC) Green Inventory” (https://www.wipo.int/classifications/ipc/green-inventory/home, accessed on 10 December 2025.) and the applicant address and business registration address were for the three regions of Beijing, Tianjin, and Hebei.

### 4.2. Network Analysis

**Figure 2:**
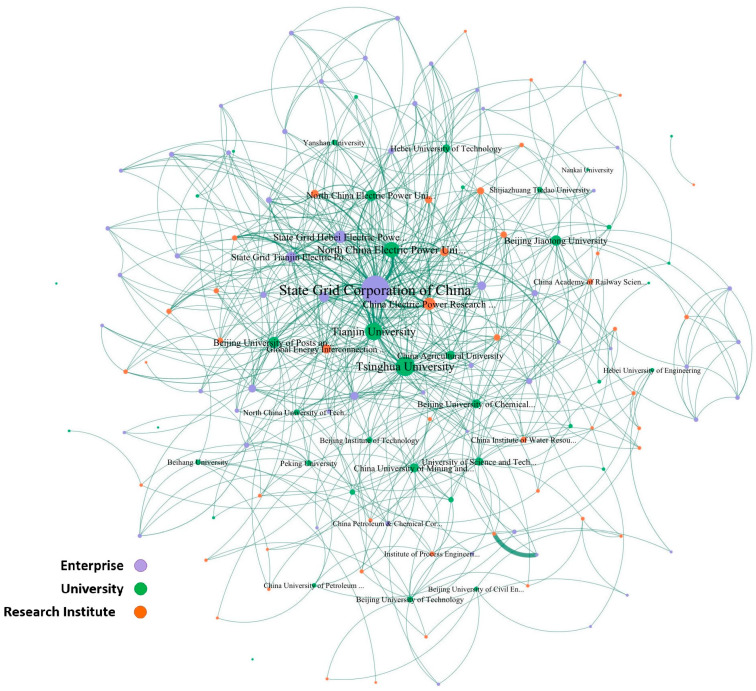
Green patent cooperation network in the Beijing-Tianjin-Hebei region.

Based on the cooperative data, the green patent cooperative network in the Beijing-Tianjin-Hebei region generated by Gephi software (v0.10.1) is shown in Figure 2. This network contains 1541 network nodes and 2246 edges. The degree of the applicant is reflected by the size of the node, and the number of cooperation times is reflected by the width of the edge. Nodes of different colors represent different levels of clustering. Observations show that the average distance between network nodes is relatively small, and the degree distribution of the nodes is very uneven, with strong heterogeneity, and there are some applicant entities with very high degrees. Compared with a random network of the same scale, it was found that this network has significantly smaller average distances than the random network and significantly larger clustering coefficients than the random network, which conforms to the small-world characteristic. In the double-logarithmic coordinate system, the degree and degree distribution of the cooperation network are approximately linear, and meet the significance requirements, thus it can be considered that the network has scale-free characteristics. The small-world and scale-free characteristics indicate that this network is suitable for using link prediction algorithms of social networks for prediction.

### 4.3. Link Prediction

This paper selects the Area Under Curve (AUC) metric to evaluate the prediction effect of the model. Compared to accuracy, recall rate, and F1, among other binary classification evaluation metrics, the unique advantage of AUC lies in its focus on ranking results rather than specific scores. Therefore, it is more suitable for evaluating ranking-type predictions. AUC is the area under the Receiver Operating Characteristic (ROC) curve. There are different calculation formulas for it in machine learning and complex networks. In this experiment, the formula based on complex networks is chosen for prediction evaluation.

In the experiment, the network edge connections are divided into the training set T1, the test set T2, and the set of non-existent edges T3. The proportion of the training set in the network edge connections is 90%, and the test set is 10%. The sampling method for T3 is as follows: construct an adjacency matrix for all nodes, and then form the T3 dataset using all node pairs (including T1 and T2) with no direct connections in the adjacency matrix. Additionally, T3 needs to maintain a 1:1 ratio with the positive samples T2 in order to avoid accuracy distortion caused by sample bias [23]. To avoid the randomness of the experiment, the random division of the training set is taken 100 times. Each division will obtain a set of similarity scores for each indicator, and the average value is taken as the final score for each indicator. Each time, a random edge is selected from the test set T2, and a random edge is selected from the set of non-existent edges T3. If the similarity score of the edge selected from T2 is greater than that of T3, then the result of this random experiment is recorded as “add 1 point”; if the two values are equal, it is recorded as “add 0.5 points”. After independent comparison for n times, the arithmetic average of the experiments is taken as the evaluation value of the precision. The specific calculation is shown in Formula (12):

(12) $$AUC=\frac{n^{\prime }+0.5n^{\prime \prime }}{n}$$

In the formula, $n^{\prime }$ represents the number of times out of n experiments that the score of T2 was higher, $n^{\prime \prime }$ represents the number of times the two were equal, and the sampling frequency n is set to 672,400 based on experience. Drawing on similar studies, when the number of sampling iterations n is set to 672,400, the absolute error of AUC can be controlled within 0.001 at a confidence level of 90%, reaching a relatively stable state. Therefore, this paper sets the number of sampling iterations n to 672,400 [24].

#### 4.3.1. Link Prediction Experiment Based on Node Similarity

First, predictions are made based on the 10 node similarity indicators given in Table 1. The results are shown in Table 4. It can be seen that the average prediction accuracy of the RA indicator is the highest (0.9171), while the average prediction accuracy of the LHN indicator is the lowest (0.8987). This suggests that the RA indicator exhibits slightly higher adaptability to this network compared to other indicators. However, the prediction values of other indicators do not differ much from this, indicating that each indicator’s prediction in this network has its merits. Therefore, this paper uses the entropy weight method to couple all 10 commonly used indicators to obtain the coupled indicators and their AUC values, and marks them in the last row of Table 3. It can be observed that the accuracy of the Coupling indicator after coupling (0.9330) is higher than that of the highest RA indicator (0.9171) among the single indicators, indicating the effectiveness of the entropy weight method in integrating indicator performance. This paper selects the coupled Coupling indicator as the node similarity indicator, and the similarity score matrix generated by this indicator is denoted as $S^{Coupling}$.

#### 4.3.2. Link Prediction Based on Path Heterogeneity

Based on the previous analysis of the green patent cooperation network structure in the Beijing-Tianjin-Hebei region, it is evident that the degree of heterogeneity of the network nodes is relatively significant. Therefore, in the prediction indicators, the role of path heterogeneity needs to be emphasized. Based on Formula (7), using the given α value, the path heterogeneity of all node queues with reachable relationships is iteratively calculated to form a path heterogeneity score matrix.

**Figure 3:**
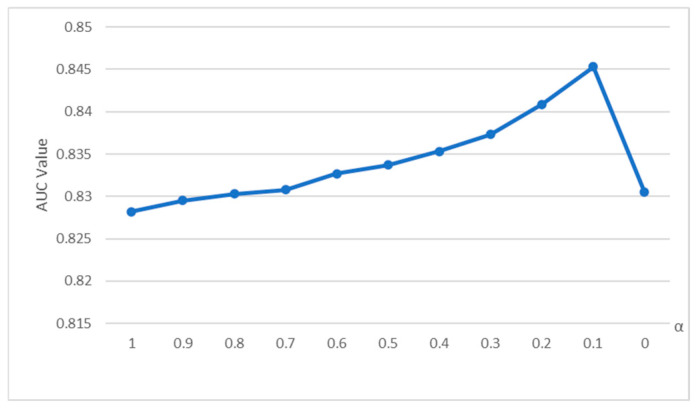
Changes in AUC accuracy of HP indicators under different α values.

To select a reasonable value for α, a sensitivity analysis was performed on α. Figure 3 illustrates the AUC accuracy of the HP indicator for various α values. It can be observed that as the α value decreases, the accuracy of the HP indicator also increases continuously, and it begins to converge when the α value is 0.1. At this time, the prediction accuracy of the HP indicator of this network is 0.8453. Therefore, the α value is set to 0.1, and the similarity score matrix generated by the HP indicator is recorded as $S^{HP}$.

#### 4.3.3. Link Prediction Based on Content Similarity

**Figure 4:**
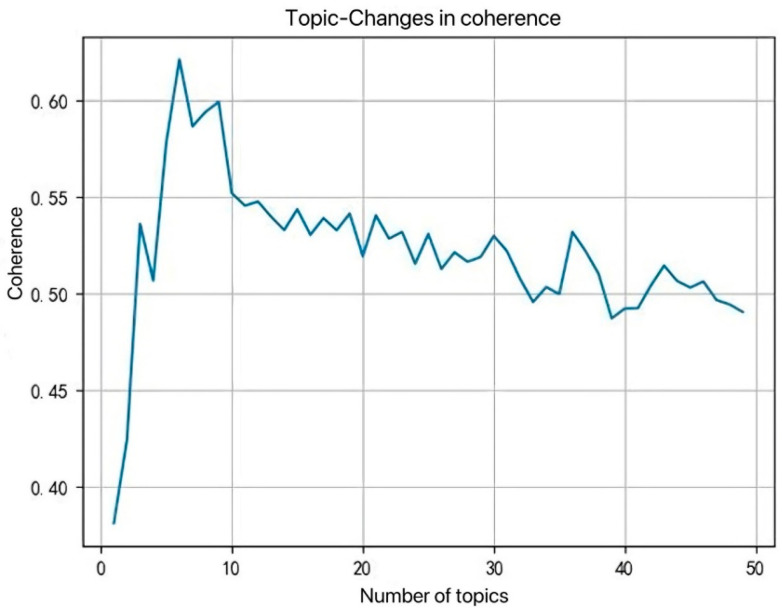
Curve chart of topic consistency.

After considering node similarity and path similarity, this section will calculate the content similarity between applicant nodes. Firstly, the patent abstracts of the training set are extracted. After tokenization processing and removal of stop words, a dictionary and corresponding corpus are established, and each word is assigned a unique number. Then, the CV consistency method is used to draw the topic consistency diagram as shown in Figure 4. The sensitivity analysis of the number of topics K reveals that the coherence is highest when K = 6, and there is a significant inflection point. Therefore, in the patent collaboration network of this paper, K = 6 is selected as the optimal number of topics.

After obtaining the number of topics, an LDA topic model is constructed. Then, the topic probability distribution of each abstract is extracted from the model, and the topic with the highest probability is selected as the final topic of the patent, forming a comprehensive dataset based on patent abstracts—patent applicants—patent topics, totaling 4318 items. For each topic, several words correspond to it, representing the technical direction of the topic, as shown in Table 5.

Then, the topic of each patent record is assigned to all applicants of the patent, and all topics linked to each applicant are aggregated to obtain the complete set of topic vectors associated with the applicant. The specific method is as follows: first, the LDA model is applied to patent texts for topic identification, generating the six aforementioned topics. Next, the topic corresponding to each individual patent is determined and assigned to the patent applicant nodes of that patent. In this way, if an applicant participates in multiple patent applications, the applicant will have a topic set—that is, a topic set vector for each network node. Then, assign the topic of each patent data to all the applicants of that patent, combine all the topics of the applicants, and obtain the set of all topic vectors associated with each applicant.

Using the six distance calculation methods listed in Table 3, the content similarity of the topic set vectors of node pairs is calculated. The AUC values corresponding to each distance are shown in Table 6. The experimental results show that the accuracy of the Jaccard, Euclidean, and Manhattan indicators is significantly higher than that of the Cosine, Pearson, and Tanimoto indicators. Therefore, these three indicators are selected, and the CS metric is calculated using Formula (9). The AUC value of CS is 0.7847, which is significantly higher than that of the single vector similarity indicator. The similarity score matrix obtained after coupling is denoted as $S^{CS}$.

### 4.4. Link Prediction Based on NPC Metrics 

After obtaining matrices $S^{Coupling}$, $S^{HP}$, and $S^{CS}$, the three are weighted to form a new prediction model, labeled as $S^{NPC}$. The weight values of $S^{Coupling}$, $S^{HP}$ and $S^{CS}$ in the new model are set as $x_{1},x_{2},$ and $x_{3}$ respectively. To obtain the optimal weights, a mathematical model with AUC of $S^{NPC}$ as the objective was established as described in Formula (11). The model is solved by means of the Grey Wolf Optimizer (GWO), and the optimal weights $x_{1}=0.9357$, $x_{2}=0.0636$, $x_{3}=0.0007$, as shown in Table 7. The result indicates that $S^{Coupling}$ has the greatest impact on the prediction accuracy, while $S^{CS}$ has the least impact. The AUC obtained under the optimal weights is 0.9627, which is significantly better than that of the individual metric of $S^{Coupling}$, $S^{HP}$, and $S^{CS}$.

From Table 7, it can also be observed that in the optimal weight combination of the NPC indicators, although the influence of $S^{CS}$ is very small, when it is included in the prediction indicators, the prediction accuracy (0.9504) is better than that considering only $S^{Coupling}$ and $S^{HP}$. This indicates that although the weight value of the CS indicator is very small, it can still significantly improve prediction accuracy by being included, highlighting the necessity of considering comprehensive indicators. Similarly, the weight value of the path heterogeneity (HP) indicator is also small, but it also improves the overall prediction accuracy.

## 5. Analysis of Link Prediction Results

**Figure 5:**
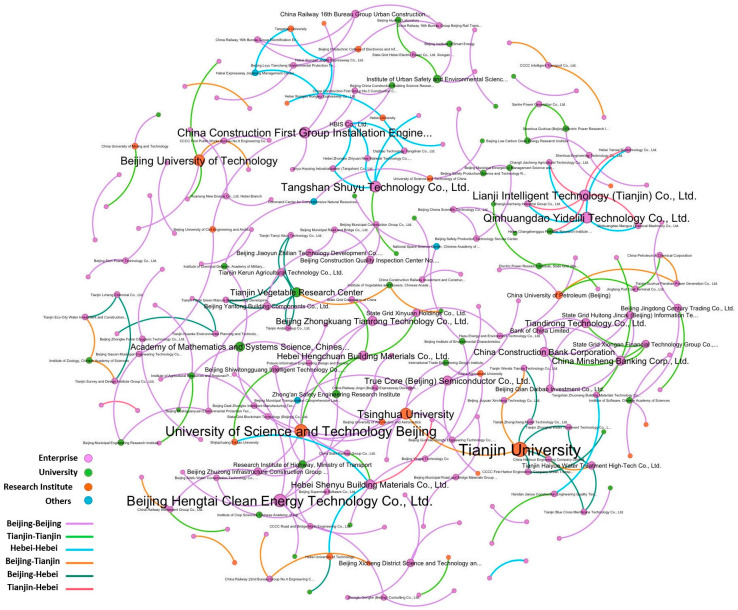
Network diagram of prediction results.

Based on the constructed novel prediction model, link prediction was performed on all unconnected node pairs in the green patent cooperation network of the Beijing-Tianjin-Hebei region. In typical new link prediction tasks, 0.1–1% of the total number of potential node pairs is usually selected to generate the predicted network [15]. An analysis of the network spanning 2020–2023 shows that the proportion of actual edges to all possible edges is 0.2%; thus, this study adopted 0.2% as the threshold. The NPC indicators were calculated for all unconnected node pairs, and the top 0.2% of node pairs ranked by NPC scores were selected to generate the predicted network. In this way, the predicted network of green patent cooperation in the Beijing-Tianjin-Hebei region was obtained, as illustrated in Figure 5. The prediction network contains 232 nodes and 219 edges. In the figure, nodes of different colors represent the main types of patent applicants; nodes of different sizes indicate the degree difference among applicants; and edges of different colors represent the patent cooperation relationships between different regions.

**Figure 6:**
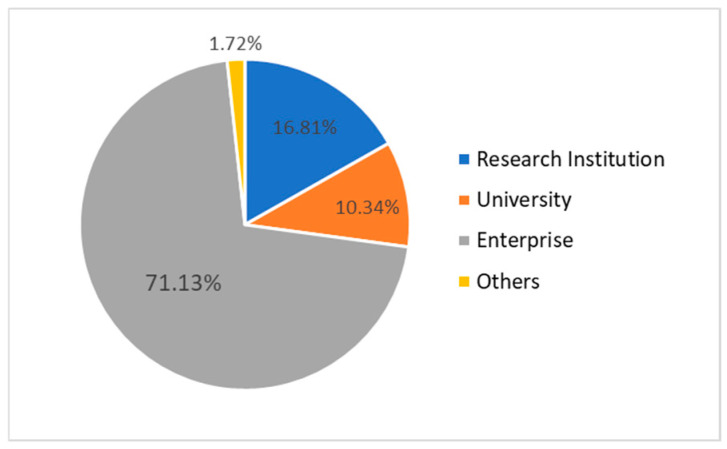
Distribution of applicant entity types in the prediction network.

The analysis of the primary type data is shown in Figure 6. It can be seen that in the prediction network, enterprises still dominate among the patent applicants, indicating that enterprises will remain the main force of innovation in the future. Such an innovative development trend not only conforms to the development law dominated by the market but also fully reflects the crucial role of enterprises in innovation. Among all the cooperation nodes, although research institutions account for about six percentage points more than universities, it can be clearly seen from the network diagram that the degree of university nodes is generally larger than that of research institution nodes, and they are also more likely to form structural holes. Therefore, enterprises and the government should pay more attention to the importance of universities in the future patent cooperation network, and make good use of the professional advantages and network intermediary role of universities. At the same time, attention should also be paid to the intermediary ability of research institutions, fully promoting the transformation of the vast knowledge resources of research institutions into productive forces and cultivating more influential research institutions.

**Figure 7:**
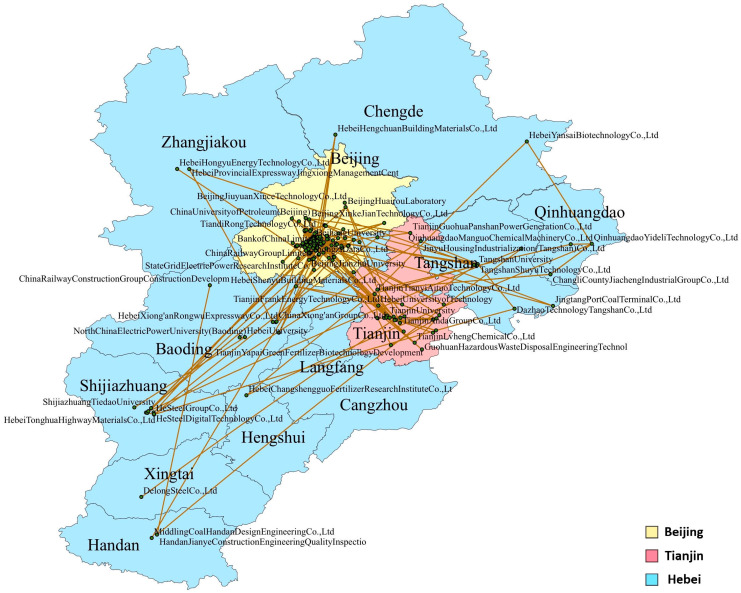
Map mapping of predicted network nodes.

By mapping the nodes in the prediction network onto the map of the Beijing-Tianjin-Hebei region, a regional distribution map of the future green patent cooperation network can be obtained, as shown in Figure 7. It can be seen that the distribution of applicants in Beijing is the densest, followed by Tianjin, and the distribution in Hebei is the sparsest.

**Figure 8:**
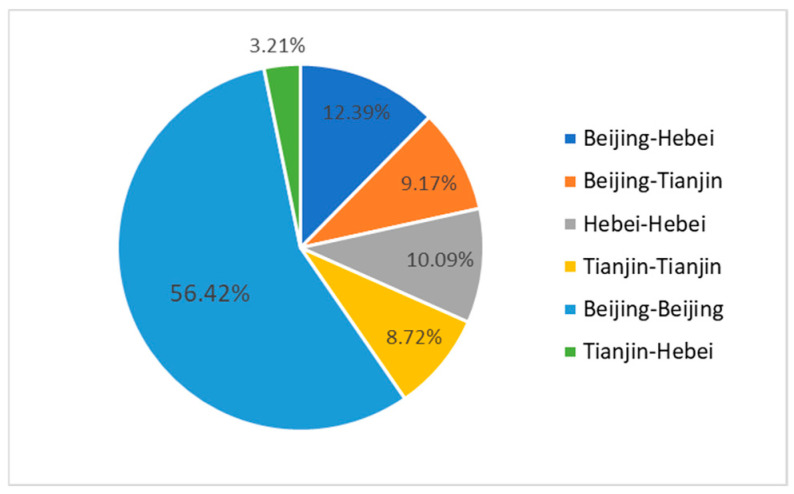
Distribution of regions with predicted connections.

The analysis of the data in Figure 7 is shown in Figure 8. It can be observed that in the future green cooperation, the number of patent cooperation within the Beijing region accounts for more than half, while cross-regional cooperation between Tianjin and Hebei accounts for only 3.21%. This indicates that Beijing’s independent innovation ability in the future remains the strongest, while the cross-regional cooperation between Tianjin and Hebei is the weakest. The patent cooperation within the Hebei region (10.09%) is higher than that within the Tianjin region (8.72%), indicating that future innovation cooperation within Hebei is likely to be better than that within Tianjin, suggesting certain innovation potential. The data in Figure 7 also indicate that a regional imbalance persists in the future innovation development of the Beijing-Tianjin-Hebei region, with cross-regional cooperation generally lower than internal regional cooperation. In terms of cross-regional cooperation, the patent cooperation between Beijing and Hebei (12.39%) is higher than that between Beijing and Tianjin (9.17%), indicating that Tianjin’s future cross-regional cooperation innovation ability still needs improvement. From the perspective of regional collaborative development, the government should promote more innovative cooperation between Beijing and Tianjin to achieve the radiation and leading role of innovation in Beijing. For instance, the government should provide special funds for Beijing-Tianjin collaborative innovation, build a Beijing-Tianjin innovation resource sharing platform, and establish a mechanism for two-way flow of talents between Beijing and Tianjin. In cross-regional cooperation, the cooperation between Tianjin and Hebei has the lowest proportion, indicating that both regions are relatively dependent on Beijing’s innovation resources, and there has not yet been a mutual driving effect in collaborative innovation between Tianjin and Hebei. To improve the situation, the government should make full use of the national-level platforms such as Tianjin Binhai New Area and Hebei Xiongan New Area, and jointly establish Tianjin-Hebei Industrial Cooperation Demonstration Parks. The parks should focus on fields with strong complementarity between the two sides such as new energy and high-end equipment manufacturing, guide upstream and downstream enterprises in the industrial chain to settle in, and promote innovative cooperation between enterprises in Tianjin and Hebei.

## 6. Conclusions

This paper, based on the green patent cooperation network in the Beijing-Tianjin-Hebei region from 2020 to 2023, established a new link prediction model. This model integrates the prediction indicators of node characteristics, path characteristics, and content characteristics, and improves each of these indicators. In terms of node similarity, the entropy weight method is used to couple 10 node similarity indicators to improve the prediction accuracy of multiple indicators in the patent cooperation network. In terms of path heterogeneity, the heterogeneity of intermediate edges and intermediate nodes in the network is simultaneously introduced into the path characteristics to construct a weighted prediction model for multi-level paths. In terms of content similarity, the LDA topic model is used to classify the patent abstracts, and the entropy weight method is coupled with the precision values of the higher accuracy Jaccard, Euclidean, and Manhattan indicators. Finally, considering the indicators of node similarity, path heterogeneity, and content similarity based on the model, a comprehensive prediction model NPC was constructed. The optimal weight values of the three-dimensional indicators were solved using GWO method which takes the AUC as the objective.

The results of the comparative experiments demonstrate that the NPC-based prediction model, which comprehensively incorporates the network’s node features, path features, and collaborative content, achieves significantly higher prediction accuracy than models relying on any single category of indicators. In the optimal weight combination of the NPC indicators, node similarity indicators contribute the most, followed by path indicators, while CS indicators make the smallest contribution. Despite their relatively low weight, CS indicators still remarkably improve the prediction accuracy, which underscores the necessity of indicator integration. An analysis of the predicted network was conducted from two dimensions: the distribution of agent types and regional distribution. The findings reveal that imbalances will persist in the future development of green technology collaborative innovation in the Beijing-Tianjin-Hebei region, and the driving effect of cross-regional collaborative innovation should be further strengthened.

Although the prediction performance of the model proposed in this paper is relatively satisfactory, there are still certain limitations and shortcomings. (1) The methodology adopted in this paper is indicator fusion, which integrates multiple mainstream prediction indicators to improve prediction accuracy. However, this method involves an excessive number of indicators, leading to extremely high computational complexity when applied to large-scale networks. Therefore, this method is not suitable for the prediction of large-scale networks. (2) Given that this method constructs a new prediction model based on indicator fusion, it is necessary to adjust the combination scheme of various indicators according to AUC during the fusion process, resulting in strong dependence on datasets. It is possible that indicator fusion may fail to improve prediction accuracy when applied to other datasets. (3) This paper only considers the link prediction of potential edges. However, practical patent cooperation networks are weighted networks, where existing cooperative connections still have the possibility of re-collaboration—in other words, the weights of edges also need to be predicted.

To address the aforementioned limitations, the future work of this study will focus on two aspects. First, more datasets of patent cooperation networks will be utilized to verify the effectiveness of the proposed method. Second, based on the weighted network data of patent cooperation, the weights of existing cooperative edges will be predicted, so as to more accurately evaluate the future evolutionary trends of the network.

## Figures and Tables

**Table 1:** Summary of the related work.

References	Methods	Model Characters	PNP
[15,17,18,19]	CN, Salton, Jaccard, HPI, LHN-I, AA, RA	Based on node similarity	
[20]	Use the indicator with the highest accuracy	Compared 10 common indicators based on node similarity	(Yes)
[21]	Integrate four most accurate prediction indicators of node similarity	Based on the entropy weight method and node similarity	√
[22]	Katz	Based on global path	
[23,24]	LP	Based on local path	
[25]	Significant Path (SP) indicator	Based on the degree heterogeneity of intermediate nodes	
[26]	The semi-local index NSI (Neighbor Set Information)	Based on the degree heterogeneity of intermediate edges	
[27]	Katz + cosine interest similarity	Based on both path and content feature	√
[28]	CN + RA + Jaccard + AA + Katz	Based on both node and path feature	√
[16]	LDA topics similarity	Based on Content similarity	
[29]	Keyword + content similarity	Based on Content similarity	√
[30]	Similarity of subject-action-object semantics	Based on Content similarity	√
[31]	LDA topics similarity	Based on Content similarity	√
[32]	Bibliographic coupling + LDA topics similarity	Based on Content similarity	√
[33]	Path similarity + topics similarity	Based on content and path similarity	
This study	Node + Path + content similarity	Based on node, path and content similarity Path similarity integrates the heterogeneity of intermediate nodes and edges	√

√ indicates that these experiments are applied in patent network prediction (PNP).

**Table 2:** Definitions of 10 classic node similarity indicators.

Indicator	Definition
CN (Common Neighbors)	$S_{xy}^{CN}=\left|\Gamma \left(x\right)\cap \Gamma \left(y\right)\right|$
Jaccard	$S_{xy}^{Jaccard}=\frac{\left|\Gamma \left(x\right)\cap \Gamma \left(y\right)\right|}{\left|\Gamma \left(x\right)\cup \Gamma \left(y\right)\right|}$
Sorenson	$S_{xy}^{Sorenson}=\frac{2\left|\Gamma \left(x\right)\cap \Gamma \left(y\right)\right|}{k\left(x\right)+k\left(y\right)}$
HPI (Higher-Order Neighbor Priority)	$S_{xy}^{HPI}=\frac{\left|\Gamma \left(x\right)\cap \Gamma \left(y\right)\right|}{min\left\{k\left(x\right),k\left(y\right)\right\}}$
HDI (Heat Diffusion Index)	$S_{xy}^{HDI}=\frac{\left|\Gamma \left(x\right)\cap \Gamma \left(y\right)\right|}{max\left\{k\left(x\right),k\left(y\right)\right\}}$
LHN	$S_{xy}^{LHN}=\frac{\left|\Gamma \left(x\right)\cap \Gamma \left(y\right)\right|}{k\left(x\right)k\left(y\right)}$
PA (Preferential Attachment)	$S_{xy}^{PA}=k\left(x\right)\cdot k\left(y\right)$
RA (Resource Allocation)	$S_{xy}^{RA}=\sum_{z\in \Gamma \left(x\right)\cap \Gamma \left(y\right)}\frac{1}{k\left(z\right)}$
AA (Adamic-Adar)	$S_{xy}^{AA}=\sum_{z\in \Gamma \left(x\right)\cap \Gamma \left(y\right)}\frac{1}{lgk\left(z\right)}$
Salton	$S_{xy}^{Salton}=\frac{\left|\Gamma \left(x\right)\cap \Gamma \left(y\right)\right|}{\sqrt{k\left(x\right)\cdot k\left(y\right)}}$

**Table 3:** Vector similarity indicators.

Indicator	Formula
Euclidean	$\sqrt{\sum_{i=1}^{n}{\left(x_{i}-y_{i}\right)}^{2}}$
Cosine	$\frac{\Sigma_{i=1}^{n}x_{i}y_{i}}{\sqrt{\Sigma_{i=1}^{n}x_{i}^{2}}\cdot \sqrt{\Sigma_{i=1}^{n}y_{i}^{2}}}$
Jaccard	$\frac{\left|X\cap Y\right|}{\left|X\cup Y\right|}$
Manhattan	$\Sigma_{i=1}^{n}\left|x_{i}-y_{i}\right|$
Pearson	$\frac{\Sigma_{i=1}^{n}\left(x_{i}-\bar{x}\right){(y}_{i}-\bar{y})}{\sqrt{\Sigma_{i=1}^{n}{\left(x_{i}-\bar{x}\right)}^{2}}\cdot \sqrt{\Sigma_{i=1}^{n}{{(y}_{i}-\bar{y})}^{2}}}$
Tanimoto	$\frac{\Sigma_{i=1}^{n}x_{i}y_{i}}{\sqrt{\Sigma_{i=1}^{n}x_{i}^{2}}+\sqrt{\Sigma_{i=1}^{n}y_{i}^{2}}-\Sigma_{i=1}^{n}x_{i}y_{i}}$

**Table 4:** AUC values of 10 node similarity indicators and AUC values of the coupling indicators.

Indicator	AUC Value
CN	0.9114
Jaccard	0.9010
Sorenson	0.9003
HPI	0.9036
HDI	0.9007
LHN	0.8987
PA	0.9013
RA	0.9171
AA	0.9168
Salton	0.9011
Coupling	0.9330 *

* Maximum value.

**Table 5:** Topic number and topic keywords.

Topic Number	Topic Keyword
1	Materials, preparation, concrete, cement, gypsum, filling, desulfurization, steel slag, aggregate…
2	Data, information, prediction, model, assessment, evaluation, system, indicator, module…
3	Steam, components, evaporator, flue gas, plate, water, device, liquid…
4	Catalyst, reaction, soil, separation, wastewater, metal, waste water, activity, purification, oxidation…
5	Voltage, current, fault, signal, circuit, battery, power, direct current, line…
6	Model, system, load, scheduling, power grid, photovoltaic, power, energy storage, distribution network, power system…

**Table 6:** AUC values of six content similarity indicators and AUC value of the CS indicator.

Vector Similarity Metrics	AUC Value
Euclidean	0.7421
Cosine	0.2845
Jaccard	0.7843
Manhattan	0.7448
Pearson	0.0065
Tanimoto	0.5061
CS	0.7847 *

* Maximum value.

**Table 7:** Comprehensive comparison of AUC values for each similarity metric.

Predictor	AUC Value			
CN	0.9114			
Jaccard	0.9010			
Sorenson	0.9003			
HPI	0.9036			
HDI	0.9007			
LHN	0.8987			
PA	0.9013			
RA	0.9171			
AA	0.9168			
Salton	0.9011			
Coupling	0.9330			
HP	0.8519			
CS	0.7847			
	$x_{1}$	$x_{2}$	$x_{3}$	AUC value
NPC	0.9388	0.0612	0	0.9504
NPC	0.9331	0	0.0669	0.9502
NPC	0	0.9988	0.0012	0.9033
NPC *	0.9357	0.0636	0.0007	0.9627 *

* Maximum value.

## Data Availability

The original contributions presented in this study are included in the article. Further inquiries can be directed to the corresponding author.

## Abbreviations

The following abbreviations are used in this manuscript:

CN	Common Neighbor
AA	Adamic-Adar
HPI	Hub Promoted Index
RA	Resource Allocation
NSI	Neighbor Set Information
MPCP	Meta-Path Computation Prediction
LDA	Latent Dirichlet Allocation
NPC	Node & Path & Content
IPC	International Patent Classification
AUC	Area Under Curve
ROC	Receiver Operating Characteristic

## References

[1] Tsay M.Y., Liu Z.W. (2020). Analysis of the patent cooperation network in global artificial intelligence technologies based on the assignees. World Pat. Inf..

[2] Chen W., Qu H., Chi K. (2021). Partner Selection in China Inter-organizational Patent Cooperation Network Based on Link Prediction Ap-proaches. Sustainability.

[3] Geum Y., Lee S., Yoon B., Park Y. (2013). Identifying and Evaluating Strategic Partners for Collaborative R&D: Index-based Approach Using Patents and Publications. Technovation.

[4] Zsolt T.K., Attila I.K., Tibor C., Beáta F. (2024). Analysis and prediction of the Horizon 2020 R&D&I collaboration network. Expert Syst. Appl..

[5] Wang J., Wang N., Zhao W., Feng L. (2025). Identifying and evaluating R&D partners via patent-based multilayer networks from the perspective of knowledge complementarity: A case study. Comput. Ind. Eng..

[6] Liu Q. Discovering of Cooperation Pattern Evolution Path in Tsinghua University: A Patent Cooperation Network Perspective. Proceedings of the 2015 2nd International Conference on Education Reform and Modern Management (ERMM2015).

[7] Liu W., Song Y., Bi K. (2021). Exploring the Patent Collaboration Network of China’s Wind Energy Industry: A Study Based on Patent Data from CNIPA. J. Renew. Sustain. Energy Rev..

[8] Mei C., Cong J., Jin S. (2019). The Characteristics and Efficiency of Innovation Cooperation of Beijing-Tianjin-Hebei Region—Evidence from Patents and Papers cooperation. J. Hebei Univ. Technol. (Soc. Sci. Ed.).

[9] Liu Y., Shao X., Tang M., Lan H. (2021). Spatial-temporal Evolution of Green Innovation Network and Its Multidimensional Proximity Analysis: Empirical Evidence from China. J. Clean. Prod..

[10] Zhou J. (2019). The Spatial-temporal Evolution and Cooperation Networks of Green Technology Innovation in the Yangtze River Delta Urban Agglomeration. Master’s Thesis.

[11] Fan J., Xiao Z. (2021). Analysis of Spatial Correlation Network of China’s Green Innovation. J. Clean. Prod..

[12] Wang K., Bian Y., Cheng Y. (2022). Exploring the Spatial Correlation Network Structure of Green Innovation Efficiency in the Yangtze River Delta, China. Sustainability.

[13] Bai Y., Wang J., Jiao J. (2012). A Framework for Determining the Impacts of a Multiple Relationship Network on Green Innovation. Sustain. Prod. Consum..

[14] Dai C., Chen L., Li B., Li Y. (2017). Link prediction in multi-relational networks based on relational similarity. Inf. Sci..

[15] Liben-Nowell D., Kleinberg J. (2007). The link-prediction problem for social networks. J. Am. Soc. Mation Sci. Technol..

[16] Chuan P.M., Son L.H., Ali M., Khang T.D., Huong L.T., Dey N. (2018). Link prediction in co-authorship networks based on hybrid content similarity metric. Appl. Intell..

[17] Martínez V., Berzal F., Cubero J.C. (2016). A survey of link prediction in complex networks. ACM Comput. Surv..

[18] Haghani S., Keyvanpour M.R. (2019). A systemic analysis of link prediction in social network. Artif. Intell. Rev..

[19] Kumar A., Singh S.S., Singh K., Biswas B. (2020). Link prediction techniques, applications, and performance: A survey. Phys. A Stat. Mech. Appl..

[20] Zhang R., Zhao Z., An M. (2021). Structure Analysis and Link Prediction of Patent Technology Cooperation Network in Guang-dong-Hong Kong-Macao Greater Bay Area. China Invent. Pat..

[21] Shi A., Qiu J., Zhao S. (2020). Patent Technology Cooperation and Link Prediction Among Science and Technology Subjects in Beijing, Tianjin and Hebei. Mod. Inf. Technol..

[22] Katz L. (1953). A new status index derived from sociometric analysis. Psychometrika.

[23] Zhou T., Lü L., Zhang Y.C. (2009). Predicting missing links via local information. Eur. Phys. J. B.

[24] Lü L., Jin C.H., Zhou T. (2009). Similarity index based on local paths for link prediction of complex networks. Phys. Rev. E Stat. Nonlinear Soft Matter Phys..

[25] Zhu B., Xia Y. (2015). An information-theoretic model for link prediction in complex networks. Sci. Rep..

[26] Zhu X.Z., Tian H., Cai S.M., Huang J., Zhou T. (2014). Predicting missing links via significant paths. Europhys. Lett..

[27] Liu J., Sun W. (2017). Discovery of potential scientific and technical collaborative relationship based on link prediction. Inf. Stud. Theory Appl..

[28] Wang Z., Han W., Sun Z., Pan X. (2019). Research on scientific collaboration prediction based on the combination of network topology and node attributes. Inf. Stud. Theory Appl..

[29] Jeon J., Lee C., Park Y. (2011). How to use patent information to search potential technology partners in open innovation. J. Intellect. Prop. Rights.

[30] Wang X.F., Wang Z.N., Huang Y., Liu Y.Q., Zhang J., Heng X.F., Zhu D.H. (2017). Identifying R&D partners through Sub-ject-Action-Object semantic analysis in a problem & solution pattern. Technol. Anal. Strateg. Manag..

[31] Kang J., Lee J., Jang D., Park S. (2019). A methodology of partner selection for sustainable Industry-University cooperation based on LDA topic model. Sustainability.

[32] Park I., Jeong Y., Yoon B., Mortara L. (2015). Exploring potential R&D collaboration partners through patent analysis based on bibliographic coupling and latent semantic analysis. Technol. Anal. Strateg. Manag..

[33] Ding J.D., Guo J. (2021). Mining potential author cooperative relationships based on the similarity of content and path. Inf. Stud. Theory Appl..

